# Chimpanzee Coordination and Potential Communication in a Two-touchscreen Turn-taking Game

**DOI:** 10.1038/s41598-020-60307-9

**Published:** 2020-02-25

**Authors:** Pavel V. Voinov, Josep Call, Günther Knoblich, Marina Oshkina, Matthias Allritz

**Affiliations:** 1 Department of Cognitive Science, Central European University, Oktober 6 u. 7, H-1051 Budapest, Hungary; 2 Department of Developmental and Comparative Psychology, Max Planck Institute for Evolutionary Anthropology, Deutscher Platz 6, Leipzig, D-04103 Germany; 3 School of Psychology & Neuroscience, University of St Andrews, St. Andrews, Fife, KY16 9JU UK

**Keywords:** Psychology, Animal behaviour

## Abstract

Recent years have seen a growing interest in the question of whether and how groups of nonhuman primates coordinate their behaviors for mutual benefit. On the one hand, it has been shown that chimpanzees in the wild and in captivity can solve various coordination problems. On the other hand, evidence of communication in the context of coordination problems is scarce. Here, we investigated how pairs of chimpanzees (*Pan troglodytes*) solved a problem of dynamically coordinating their actions for achieving a joint goal. We presented five pairs of chimpanzees with a turn-taking coordination game, where the task was to send a virtual target from one computer display to another using two touch-screens. During the joint practice of the game some subjects exhibited spontaneous gesturing. To address the question whether these gestures were produced to sustain coordination, we introduced a joint test condition in which we simulated a coordination break-down scenario: subjects appeared either unwilling or unable to return the target to their partner. The frequency of gesturing was significantly higher in these test trials than in the regular trials. Our results suggest that at least in some contexts chimpanzees can exhibit communicative behaviors to sustain coordination in joint action.

## Introduction

Joint coordinated action constitutes a prominent and distinctive aspect of social cognition. The ability to coordinate joint actions through communicative means has been available to human groups and, hypothetically, was available to its immediate ancestors from *Homo* genus^1–3^. By coordinating their actions individuals can achieve things they would never be able to achieve alone. In this way joint actions are a major building block of human culture^4^. Therefore, it is a crucial question which precursors of joint action coordination are already present in other species and which joint action skills (if any) are only present in humans. In the present study we investigated the joint action coordination abilities and their social-cognitive underpinnings in one of our closest phylogenetic relatives, chimpanzees (*Pan troglodytes*).

Chimpanzees in their natural habitats constantly face coordination and cooperation problems, such as hunting together^5–9^, teaming up with others during conflicts^10,11^ or simply traveling together^12,13^. Several experimental studies in captivity have demonstrated that chimpanzees can solve various coordination problems^14–20^. Common to these experimental studies is the finding that chimpanzees either don’t use communication to coordinate^18,21^, or that communication for coordination is rare^14,15^. Although recently Melis & Tomasello^22^ reported that pairs of chimpanzees established a successful coordination by communication, they did not observe any gestures between partners. Instead, information about the tool location was primarily transferred by the informed individual approaching the target box. Thus, it is unclear whether chimpanzees were communicating or whether the uninformed partner used the position of her partner to decide which box to open. This is in a sharp contrast to human children, who readily employ communication to solve similar coordination problems starting from an early age^15,23,24^. A relevant finding in this context is that children’s abilities to coordinate with peers are also correlated with language proficiency^25^, indicating that there is a link between language and joint action capabilities^26^.

Although this may be taken to suggest that chimpanzees have low communication abilities in general, the apparent lack of evidence for communication for joint action coordination does not reflect a lack of communicative abilities. Chimpanzees, among other primate species^27,28^, communicate different kinds of information in the wild^29–31^ and in captivity^32–36^. In the wild, chimpanzees use vocal communication to warn others about the presence of potential threats^37^, modulate social dynamics^38^, communicate about food^39^, and to greet and indicate social status^40^. They also use gesturing to initiate travel with infants^13^, and solicit play^14^ or sexual attention^30^. In addition, in captivity, they use gestures to request tools from conspecifics^41^ or to request instrumental actions to help them to achieve their goals^42^, although their gestural communications seems to be limited to proto-imperative requests, such as demanding an object or a self-directed action^30,43^. There is converging evidence that chimpanzees are flexible and adaptive to the context in the production of their gestures^34,43–46^. Furthermore, sign language studies with cross-fostered chimpanzees have demonstrated that they can adaptively use symbolic communication (by repeating or modifying their utterances) to repair conversational breakdowns^47^. Several studies have suggested that gestures and vocalizations can be used in great apes to modulate (or coordinate) social interactions^29,45,48^, such as play behavior or grooming^49^. Taking these facts together, it is puzzling that these varied communicative abilities are rarely utilized in a situation of *joint action*, where two or more individuals need to coordinate their individual actions for achieving the joint goal.

In this study, we developed a novel approach to investigate the role of communication for joint action coordination in non-human primates: Chimpanzees learnt to play a turn-taking game, where the shared goal was to move a virtual target from one computer screen to another. We used the task to address two questions. First, we asked whether pairs of chimpanzees would spontaneously employ communicative means to achieve the joint goal, although coordination could equally be achieved without communication. Second, we manipulated the task to simulate a coordination break-down to test whether communication is more likely to occur when coordination fails. We highlight that our approach is different from previous research on coordination based on social dilemmas^15–17,23,50^ in two important aspects.

First, our task required continuous turn-taking interaction rather than a one-shot decision or a series of decisions where an individual has time for deliberation. Previous research has demonstrated that chimpanzees spontaneously take turns to coordinate in a task previously learnt individually and adapt their actions to their partner’s rhythm^51^. Similarly, turn-taking and role alternation is a common rule for young children’s games with adults and peers. From their third year of life, children can coordinate taking turns with peers for achieving joint goals^52^, and they start communicating to repair coordination in turn-taking interactions with adults by the end of their first year of life^53^.

Second, our task required coordinating dynamic physical actions. Classic decision dilemma tasks, such as a stag-hunt game^15,16^, require subjects to learn or understand the dependencies between one’s and a co-player’s decisions, and to take the perspective of a co-player by means of domain-general cognitive mechanisms. In contrast, coordinating joint action may utilize simulations based on an internal model of one’s own actions^54,55^ for predicting the outcome of another’s actions. Similarly, perception-action links may be used to infer another’s action goal or to use the shared goal to form such predictions^54^. Thus, coordination of joint action may build upon different, specialized cognitive capacities, possibly available to non-human primates as well^56^. These two features could increase the chance that chimpanzees spontaneously learn how to coordinate in the task and that they perceive the task as a joint coordination problem.

## Task Overview

**Figure 1:**
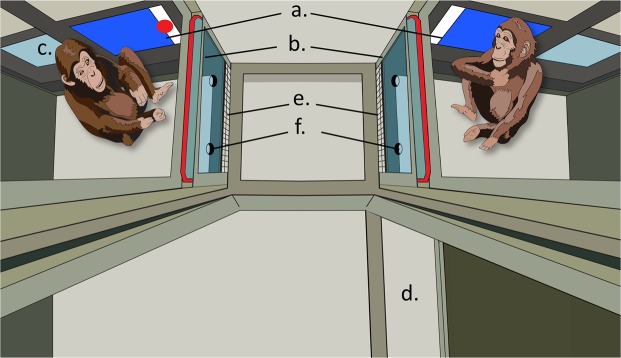
A Bird’s eye view on the experimental set-up in joint trials. (**a**) Displays with stimuli behind touch-frames. (**b**) Transparent side panels. (**c**) Position for the second display during Individual training. (**d**) Closable doorway between two compartments. (**e**) Mesh-grids under the side panels. (**f**) Holes in the side panels (used by subjects in gesturing).

The experiment involved a turn-taking coordination game, previously used in human research on joint action coordination^55^. Two chimpanzees manipulated the direction and velocity of a virtual target by interacting with it on two touchscreens (each chimpanzee was responsible for moving the target on one of the screens). To receive a food reward, the two chimpanzees needed to move the target from one screen to another several times in a row (see Fig. 1). Absence of engagement or inattentiveness to the target resulted in the target vanishing from the screen. Thus, there was no food reward on trials where the two chimpanzees did not work on the task together.

### Joint test condition

While practicing joint performance of the task, some individuals exhibited specific visual and audible behaviors, such as clapping, knocking against polycarbonate panels and reaching through mesh in the direction of their partner’s location (detailed descriptions of behaviors included in the analysis can be found in Supplementary Table S1; see also Supplementary Video S04 which contains recordings of some exemplar behaviors). Such requesting behaviors, have previously been reported to occur in the context of interactions with humans or conspecifics, and have been identified as gestures^27,36,43^. Previous research has also demonstrated that in other contexts production of such gestures in chimpanzees satisfy the criteria for intentional communication^32^. To investigate whether chimpanzees would use communication (particularly gestures), broadly defined as transmitting information to a recipient^57^, to maintain coordination, we tested: (1) whether behaviors of this type would occur more often when coordination breaks down, and (2) whether it would be exhibited more often when there is in fact a partner to work with. We emphasize that the scope of this study, its focus, and the rarity of gestures (see Results) did not allow us to study the pragmatics and the (potentially) *intentional* communicative nature of these behaviors (which is characterized by sensitivity to the partner’s attentional state, persistence and elaboration when partner remains unresponsive etc., see^29,32,34,47,57–59^). Instead, we relied on the gestures described in the literature^35,60–62^ to identify communicative behavior and measure its rate. Throughout Methods and Results we will refer to these observed behaviors as “gestures”.

**Figure 2:**
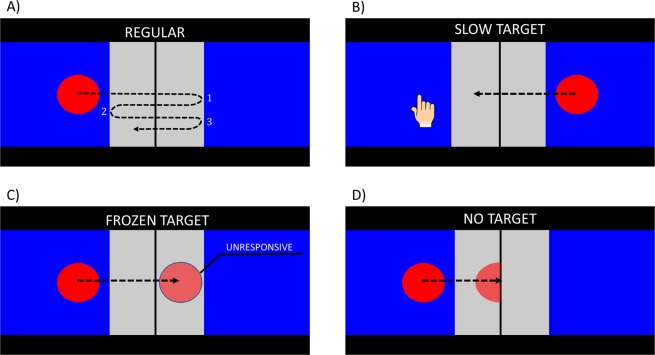
Conditions implemented at different stages of the experiment. The trial could be initiated on either screen (always the left side in this Figure). (**A**) Regular trial in Joint Training. The target was moved horizontally and needed to be stopped and sent back to another screen three times, at a constant vertical position. Upon the fourth crossing of the border between the screens, rewards were delivered. (**B**) Slow Target trials (Individual Test). The target appeared on the remote screen after trial onset and slowly moved towards the subject’s screen. (**C**) Frozen Target trials (Joint Test). Upon entering the other screen, the target became unresponsive to touches. (**D**) No Target trials (Joint and Individual Test). After leaving one screen, the target did not reappear on the other screen.

To test the second question, that is, whether the observed gesturing behavior was produced to maintain action coordination, we introduced probe trials which simulated a coordination breakdown. Since previous studies have shown that chimpanzees may take into account whether their partner shows signs of being able and/or willing to help^63^, we used two different types of probe trials (Fig. 2C,D). In the *Frozen Target* condition, after the target entered the awaiting subject’s screen, it stopped and became unresponsive to touches for 15 or 30 s. The subject faced with this target was expected to keep interacting with the target, appearing *willing but unable* to return the target to the partner. In the *No Target* condition the target vanished while moving from one screen to the other. Here, the subject should have not interacted with the screen, thus appearing *able but unwilling* to return the target to the partner.

Our main prediction was that subjects should produce gestures more often in probe trials – as these simulated a coordination breakdown – than in regular trials. For this comparison, we did not distinguish between the type of probe trial and between the role each subject had (that is, whether they were the subject appearing “unwilling/unable” or the tricked partner). This analysis is described in the main text. Specific hypotheses regarding gesturing in the two different types of probe trials under different condition of visual access to the partner’s workspace(see Methods) along with their statistical tests can be found in the Supplementary Information.

### Individual test condition

To test whether subjects would primarily exhibit gestures when there was a partner to communicate *with* – rather than frustration or impatience induced by having to wait for the target to return to their screen – subjects completed an individual test condition, which also included regular and probe trials. In regular trials subjects moved the target from one screen to the other and then immediately received a reward. The general setup was very similar to the joint test condition; the subject sat in front of one screen in one room while the second screen was in the adjacent room. As in the joint test condition, there were also two different types of probe trials (Fig. 2B,D). *Individual No Target* probe trials contrasted *No Target* probe trials from the joint test condition to assess how partner presence would affect the frequency of gestures. In these *individual No Target* probe trials the target did not appear on the other screen, and subjects had to wait for 15 or 30 s for the next trial. *Individual Slow Target* probe trials were introduced to contrast regular joint trials. In slow target probe trials, after initiation of a trial, the target started slowly moving from the other screen towards the subject’s screen. As such, these trials simulated (without a partner) the potentially frustrating situation in which a subject had to wait for their partner to slowly return the target. We reasoned that if the subjects’ gestures reflected more than general frustration, but rather a behavior intended to be perceived by a partner, there should be more such behaviors in joint regular and joint probe trials than in these potentially frustrating Individual Slow Target probe trials.

To identify gestures, we coded the videos from the experimental sessions (see Supplementary Table S1 for the complete list of gestures and their definitions). All statistical analyses were performed on the level of individual *turns*, where a turn was characterized as the time the target spent on one screen before half of it had left the screen (either to appear on the other screen or to disappear outside the playing field). Turns from trials in which the target was automatically stopped or manually stopped by the experimenter (see Methods section) were excluded from the analyses.

## Results

### Prevalence of spontaneous gesturing in the joint training condition

Table 1 shows for each subject the total number of valid turns (one successful trial required four turns in the final training step) and the proportion of turns in which at least one audible gesture, at least one visible gesture or at least one audible and at least one visible gesture (typically in succession) occurred.

**Table 1:** Total number of valid turns completed and proportion of turns with gestures in the Joint Training Condition.

Subject	Turns (total)	Turns with gestures			Proportion of turns with gestures		
		audible	visible	both	audible	visible	both
Kara	1024	7	0	0	0.0068	0	0
Fraukje^*^	1024	2	1	0	0.0020	0.0010	0
Fraukje^**^	2534	3	0	0	0.0012	0	0
Robert	2534	11	0	0	0.0043	0	0
Sandra	1355	56	42	1	0.0413	0.0310	0.0007
Tai	1355	320	1	8	0.2362	0.0007	0.0059
Bangolo	1388	17	4	0	0.0122	0.0029	0
Lome	1388	63	7	5	0.0454	0.0050	0.0036
Lobo	970	3	1	0	0.0031	0.0010	0
Kofi	970	1	1	0	0.0010	0.0010	0

^*^as paired with Kara, ** as paired with Robert.

Note that some subjects began with joint training and some subjects completed joint training after individual training (see Supplementary Information for details).

The total number of valid turns differed between subjects as a result of the different numbers of joint training sessions administered to them (see Supplementary Information), ranging between 970 and 2534 turns per subject. There was large inter-subject variability in gesturing rates. Specifically, five of nine subjects displayed gestures in less than 1% of turns. For the remaining subjects, gesturing rates ranged between 1.51% and 24.28%.

### Gesturing in regular vs. probe turns of the joint test trials

**Figure 3:**
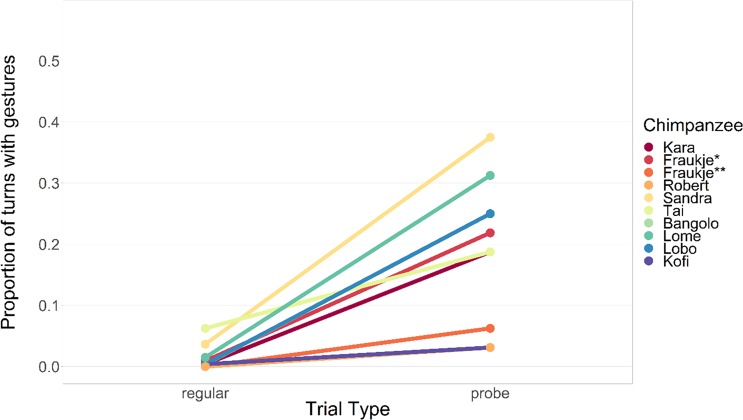
Gestures in regular and probe turns of the Joint Test condition. Note: *as paired with Kara, ** as paired with Robert.

Figure 3 shows for all subjects the proportion of Joint Test regular turns that included at least one gesture, as well as the proportion of Joint Test probe turns that did.

For all subjects gestures in regular turns were very rare (mean of individual mean proportions of turns with gestures: *M* = 0.0150, *SD* = 0.0211), whereas gestures in probe turns occurred more frequently (mean of individual mean proportions: M = 0.1962, SD = 0.1167). Note that one subject (female Fraukje) completed the Joint Test condition twice, with two different partners (see Methods).

As can be seen in Table 2, eight of nine individuals exhibited more gestures in probe turns than in regular turns of the joint test condition. To assess whether the chimpanzees gestured more frequently in the probe trials at the group level, two generalized linear mixed models (GLMMs) were fitted, each excluding one of the pairs that shared a subject. The effect of turn type on the group level was significant (Model 1, excluding pair “Fraukje & Kara”: *N* = 8, β = 2.05, Χ^2^(1) = 6.34, *p* = 0.012; Model 2, excluding pair “Fraukje & Robert”: *N* = 8, β = 1.92, Χ^2^(1) = 6.50, *p* = 0.011). In addition, there was a positive effect of turn duration. Gesturing was more likely to occur during turns that took longer (p < 0.025 for both models). Complete model output tables can be found in the Supplementary Materials. An analysis that compared the two types of probe-trials (No Target vs. Frozen Target) can also be found in the Supplementary Materials.

**Table 2:** Differences in gesturing frequency for each individual between Joint Test regular turns and Joint Test probe turns.

	Sign of difference	Firth logistic regression				Χ^2^-test	
		β	*SE*	Χ^2^	*p*	Χ^2^	*p*
Kara	+	4.28	0.74	31.68	<0.001	76.37	<0.001
Fraukje^*^	+	3.33	0.55	28.44	<0.001	65.95	<0.001
Fraukje^**^	+	4.26	1.23	11	0.001	19.04	<0.001
Robert	+	3.86	1.27	6.26	0.012	3.9	0.048
Sandra	+	3.03	0.49	36.24	<0.001	56.26	<0.001
Tai	+	1.6	0.53	7.88	0.005	5.35	0.021
Bangolo	+	4.69	0.77	43.02	<0.001	103.21	<0.001
Lome	+	3.44	0.52	39.09	<0.001	82.14	<0.001
Lobo	+	4.42	0.78	38.52	<0.001	88.43	<0.001
Kofi	+	2.15	0.97	3.15	0.076	0.64	0.423

^*^as paired with Kara, ^**^as paired with Robert.

### Gesturing in no target probe turns of the joint vs. individual test trials

We hypothesized that if the gesturing behaviors reported in the previous section were indeed communicative in nature, they should occur more often in situations when subjects are performing the task together with a partner rather than performing it by themselves.

**Figure 4:**
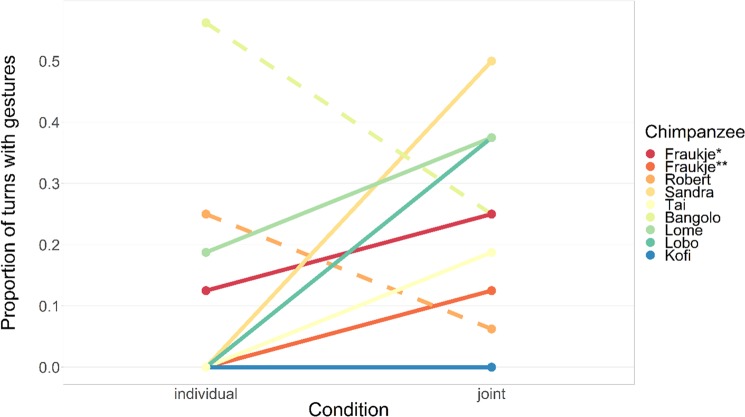
Gestures in No Target probe turns. Dashed lines represent individuals with gesturing opposite expected pattern. Note: Kara did not receive Individual Test condition, *as paired with Kara, **as paired with Robert.

For the eight subjects who completed both the Individual and Joint Test conditions, a total of 34 turns with at least one gesture were recorded for the Joint Test condition as opposed to 18 such turns in the Individual Test, out of a total of 144 turns in each of the two conditions. Figure 4 displays the total proportion of probe turns with gestures for each subject. For two subjects who either did not complete the Individual Test condition (female Kara) or who showed no gesturing in either Test condition (male Kofi), no individual inferential statistics were computed.

Table 3 presents inferential statistical results for all subjects. Two subjects (female Sandra and male Lobo) exhibited a significant difference in the rate of gesturing in the expected direction (more gestures in Joint probe turns without target), while for one subject (male Bangolo) results from the GLM and Firth logistic regression indicated a significant difference in the opposite direction (see also Supplementary Table S7). For the remaining five subjects, differences did not exist or were too subtle to reach conventional levels of statistical significance. Numerically, one subject never showed gestures in these trials, and one subject displayed more such behaviors in the Individual condition, while three displayed more gestures in the Joint Test turns. In sum, there was a weak overall tendency for subjects to gesture more in the joint condition (proportion of turns with gestures: *M* = 0.22, *SD* = 0.17) than in the individual condition (*M* = 0.15, *SD* = 0.21). However, on the group level this effect was not significant (Model 1, excluding pair “Fraukje & Kara”: *N* = 8, β = 1.12, Χ^2^(1) = 1.60, *p* = 0.205; Model 2, excluding pair “Fraukje & Robert”: *N* = 7, β = 1.35, Χ^2^(1) = 2.35, *p* = 0.125). There was a positive effect of session, gesturing was more likely to occur in later sessions within the same view block (p < 0.036 for both models). Complete model output tables can be found in the Supplementary Materials.

**Table 3:** Differences in gesturing frequency between Joint and Individual Test condition in No Target probe turns.

	Sign of difference	Firth logistic regression				Χ^2^-test	
		β	*SE*	Χ^2^	*p*	Χ^2^	*p*
Fraukje^*^	+	1.03	0.97	1.3	0.255	0.21	0.651
Fraukje^**^	+	1.63	1.4	1.41	0.236	0.53	0.465
Robert	−	−1.54	1.14	2.15	0.143	0.95	0.330
Sandra	+	3.46	1.52	9.43	0.002	8.17	0.004
Tai	+	2.73	1.75	3.46	0.063	1.47	0.225
Bangolo	−	−2.06	1.02	5.51	0.019	2.07	0.150
Lome	+	0.78	0.8	1.1	0.295	0.62	0.432
Lobo	+	4.02	1.86	8.59	0.003	5.13	0.024

^*^as paired with Kara, ^**^as paired with Robert.

### Gestures in regular turns in joint test vs. slow target probe turns in individual test

As a second comparison to test the hypothesis that gestures are more frequent when a partner is present, we compared the frequency of turns that included at least one gesture between turns in Joint regular trials (between 413 and 711 turns per pair), and in Individual Slow Target trials (16 turns per subject). Two of eight subjects who participated in joint and individual test sessions never displayed gestures in either Joint Test regular turns or Individual Test Slow Target probe turns. The proportion of gesturing in regular turns from the Joint Test condition for the remaining six subjects was very low (*M* = 0.018, *SD* = 0.023), ranging between 0.18% and 6.30%. None of these subjects ever displayed gestures in any of the Slow Target probe turns. This low rate of gestures, together with imbalanced numbers of turns available in the two conditions resulted in unreliable estimates of parameters and GLM(M) convergence issues, therefore no inferential statistics are reported here.

## Discussion

The current study presented a group of captive chimpanzees with a dynamic, two-touchscreen joint action task that required pairs to coordinate for mutual benefit. Some of the subjects occasionally exhibited visible and audible behaviors during a joint training period that have been classified as communicative gestures in other studies^34,58^. The experiment that followed established that such behaviors occurred significantly more often in situations in which coordination broke down. In addition, some of the subjects showed more gesturing when paired with a partner than when playing alone. Considered in combination, we regard these results as evidence that at least some of the tested chimpanzees communicated to coordinate with their task partner.

Previous experimental methods for studying great ape coordination have been designed to implement characteristics of specific social dilemmas borrowed from game theory, such as the Stag Hunt^15,16^ or the Snow Drift^64^. Only a handful of coordination studies have previously reported communication as coming to the rescue when coordination was about to fail^15,16,65^. Unlike these experimental studies, which observed coordination failures (and occasional communication) only as a consequence of subject’s spontaneous behaviors, in our experiment we disrupted an otherwise successful coordination process by introducing probe trials in which a partner appeared to be unwilling or unable to perform its part of the game. Under these circumstances, communication was more frequent than during regular joint test trials where we did not interfere with the joint task performance.

A second difference between our study and many previous studies is that it included a non-social control condition that tested subjects individually under circumstances comparable to coordination breakdown, but without a social partner. While it is plausible that imperative communication may sometimes be motivated by negative emotions, nonsocial control conditions are necessary to determine whether behavior that occurs during a coordination breakdown may be purely a sign of frustration over food not received. Five of eight participants displayed more communicative behaviors in probe trials of the Joint Test, compared to the Individual Test condition, and for two individuals this difference was significant, while only two out of eight individuals showed a reverse pattern. The youngest of these two individuals, and in our sample (male Bangolo), produced audible gestures (hand clapping) in multiple probe turns of the Individual test condition, indicating perhaps frustration or impatience as the primary driver of this otherwise functionally ineffective action. These findings may be viewed as allowing only very tentative conclusions at this point, but they emphasize how important it is that studies of communication for coordination include non-social control conditions whenever possible. Such manipulations should be aimed at minimizing *all* opportunity for social interaction (e.g. by removing not only the primate partner but also all human experimenters from non-social control conditions), and at maximizing the statistical power to detect differences for rare behaviors such as communicative acts.

Our findings share an important feature with the previous primate coordination studies. Chimpanzee communication in coordination experiments has so far been rare, at least rarer than for humans. The rates of chimpanzee communication in coordination experiments is typically so low that it is negligible or not reported^17,23,64,66,67^. When it was found, it was predominantly exhibited at low rates and/or only by a handful of individuals^15,16,21,65^.

The fact that communication is so rare is not readily explained by proposing that chimpanzees do not recognize the utility of communication in *any* cooperation context, as captive chimpanzees have been shown to communicate to solicit help in different experimental tasks^41,68^. Besides help solicitation, Melis and Tomasello^22^ have recently reported that chimpanzee informants communicated to their partners which box contained a set of hidden tools required to jointly extract food from an apparatus by approaching the right box, and in some cases placing the key opening the box near it. Our study complements these findings in two aspects. First, our study evades the debatable question whether cueing an object by approaching it can be qualified as communication. Second, unlike in our task, the task used by Melis and Tomasello required a successful act of communication as a necessary condition for collaboration to succeed. At the very least, our study adds that chimpanzees under certain circumstances (e.g. in trials of the Joint Training phase) would also resort to communication, even if it is not a prerequisite for successful collaboration. At the same time, as our study shows, communicative behavior is more likely to occur in a situation of imperfect coordination or when coordination breaks down. This might explain, why it has not been observed in previous studies where coordination was uninterrupted and could be perfectly achieved through perception and action (for example, in a study by Martin *et al*.^51^).

Studies that demonstrate how nonhuman primates communicate in the service of coordination remain extremely rare. This consistent picture presents us with a puzzle that will need to be addressed in future studies: Are there any circumstances that will elicit communication for coordination in chimpanzees that are similar to those observed in humans? Potential factors that may modulate the likelihood that chimpanzees use communication for joint action coordination are the 1) coordination task to be performed (e.g. how much is at stake, how much attentional focus the task demands from each partner, to what extent each subject can monitor their partner’s success, etc.), 2) the types of communication the coordination task affords (e.g. proximal vs. distal), 3) individual communicative propensities, and 4) social tolerance between partners^69^. Or is communication for coordinating action in chimpanzees bound to be rare and occurs only in response to a coordination attempt that has failed^16,69^ ? Does the rare use of communication reflect a failure in recognizing its utility or is communication simply ineffective in bringing collective benefits for coordinating partners?

An important question that the present study cannot fully answer is to what extent chimpanzee gesturing in our study constituted *intentional* communication. As explained earlier, our aim was to establish a new dynamic coordination task and to investigate whether chimpanzees would communicate when it broke down. For this purpose, we adapted specific behaviors from studies that had been shown to be intentional communicative gestures (e.g. clapping, pointing, knocking), and hypothesized that these very same behaviors, would be used more often when coordination broke down and more often when there was a partner. However, our study did not test whether chimpanzee gesturing met all the criteria for *intentional* communication. For example, while we attempted to test – based on the joint vs. individual test comparison – whether communication was sensitive to the *presence* of a partner, our behavior coding did not differentiate whether communication rates were sensitive to the partner’s *attentional state*. Further, the relatively rare occurrence of gesturing did not allow us to investigate persistence and elaboration of gesturing as a reaction to a non-responsive partner, as is sometimes done in studies of intentional gestural communication, both in captive chimpanzees^47,58^ and in wild chimpanzees^29,59^.

Since communication for coordination is so central in discussions of the evolution of collaboration in humans^15,16^, future studies of nonhuman primates should increase efforts to further tighten the link between the study of joint action coordination on the one hand^70,71^ and the study of nonverbal communication pragmatics on the other^58^. Such cross-disciplinary studies may investigate, for example, whether the production of behaviors as we observed in our study are marked by indicators of *intentional* communication. For instance, one could investigate whether such behaviors, if exhibited, are in fact comprehended by the addressee and prompt them to increase their efforts to repair coordination when addressed during a coordination event that is about to fail. Only by looking at the problem of communication for coordination in non-human primates from these different perspectives might we find eventually which cognitive capacities have made our species distinct in its ability to do things together.

In sum, we designed a dynamic task to elicit communication between pairs of chimpanzees engaged in a coordination turn-taking task. Chimpanzees spontaneously communicated with each other, a behavior that we neither trained nor shaped. We simply created suitable conditions for its appearance. Moreover, the differences between the Joint and Individual Test suggests that an interpretation of the subjects’ gestures as mere displacement behaviors or signs of frustration is incomplete. Nevertheless, communication was rare and reactive rather than frequent and proactive – mainly aimed at repairing rather than preventing a breakdown in coordination. Future studies should include non-social control conditions and a sufficiently large number of probe trials to conclusively eliminate alternative (non-communicative) explanations – such as frustration – and to accommodate for low base rates of gesturing, respectively. A fully automated testing protocol that eliminates visual access to the experimenter may further help in distinguishing displacement behaviors from genuine communicative gestures. The obvious next step will be to disentangle what chimpanzee communication for coordination, when it occurs, is *about*. Additional manipulations to coordination breakdown situations (e.g. their duration, putative responsibility, putative motivation to cooperate or defect) and in-depth analysis of individual behaviors (e.g. response waiting, attention monitoring, persistence and elaboration) will be needed to describe the pragmatics of communication for coordination, as has already been done in other domains.

## Methods

### Subjects

Nine chimpanzees, including four females, participated in this study, the age range was 6 to 39 years (*M* = 18.33, *SD* = 12.51). Further details can be found in Supplementary Tables S2A and S3A. All subjects were housed together at Leipzig Zoo, Germany, at the time the study was conducted, in semi-natural indoor and outdoor enclosures that include climbing structures, ropes and platforms. Their regular diet consists of vegetables, fruit, and occasionally cooked meat and eggs. Subjects are also provided with enrichment food items intended to stimulate extractive foraging behaviors. All research is non-invasive, and subjects are never deprived of food or water and have access to water ad libitum during testing. All research was conducted in the observation rooms at Leipzig Zoo, and all participation in testing was voluntary. Animal husbandry and research comply with the “European Associations of Zoos and Aquaria Minimum Standards for the Accommodation and Care of Animals in Zoos and Aquaria”, the “World Association of Zoos and Aquariums Ethical Guidelines for the Conduct of Research on Animals by Zoos and Aquariums” and the “Guidelines for the Treatment of Animals in Behavioral Research and Teaching” of the Association for the Study of Animal Behavior. The research was approved by the ethics joint committee of the Max Planck Institute for Evolutionary Anthropology and Zoo Leipzig (consisting of the director of the WKPRC, the research coordinator, the zoo veterinarian, the head keeper, and the assistant head keeper of great ape husbandry at Leipzig Zoo), and the methods were carried out in accordance with the aforementioned guidelines and regulations.

### Apparatus

Tests were run using a Windows PC with Intel Core 2 Duo 3.17 GHz CPU, 4 Gb of RAM, and AMD Radeon HD 6670 dedicated graphics adapter. The stimuli were prepared in Matlab (Mathworks) and presented on two ViewSonic VG930m 19″ displays with 5:4 aspect ratio, set to 1024 ×768 resolution and 60 Hz refresh rate. The displays were installed behind the openings in the wall of the testing room (see Fig. 1). Touch was recorded by means of 19″ infrared touch frames with 5:4 aspect ratio, models Nexio NIB-190B (used only during some individual training sessions) and Keytec OPTIR Touch PPMT-IR-019EL. The empty frames were embedded into metal sheets together with a layer of transparent acrylic. These panels were installed in front of the displays so that touches were performed on an acrylic surface on top of the display. There was a 5 mm gap between the display screen and the acrylic surface. The touch frames were spatially calibrated in each session, so that coordinates on the acrylic closely corresponded to the projected coordinates on the display behind. A reward beep was produced from stereo speakers installed on the floor facing each subject’s compartment.

All trials were videotaped with a GoPro3 camera providing a bird’s eye view on the set-up and two Panasonic digital cameras. The latter two cameras were directed on individual displays through the side panels.

### Materials and stimuli

The main element of the task was a red circular target, 7 cm in diameter, that could move horizontally between the two screens. The vertical position of the target was centered throughout, there never was a vertical movement of the target. The background behind the target consisted of two regions: an outer blue region (70% of the area) and an inner white region (see Fig. 3). Black boundaries (line width 1 cm) separated the active screen area from the bottom and the top.

### Procedure

The task was to repeatedly send the target back and forth between the two screens by touching it. Touches on the left screen resulted in acceleration of the target to the right and touches on the right screen resulted in acceleration of the target to the left.

#### Group A

A first of two groups of subjects (see Supplementary Table S2A,B) was initially trained to perform the task individually. During individual training the two screens were installed next to each other within a single room so that an individual subject could perform the task using both screens. Subjects were able to perform the task without physically moving between the two screens by locating themselves in between the screens (see Supplementary Video S01). During training subjects were encouraged to approach the displays and to interact with the touchscreens.

The procedure of a trial during the individual training phase was as follows. Subjects initiated a trial by touching the initiation symbol centrally presented on the otherwise blank screen. The touch made the target appear at the center of the screen. A touch upon the target initiated its linear motion towards the outer border of the adjacent screen. The second touch on the target doubled its speed. Once the target entered another screen, touches on the target first decreased its speed stepwise and then accelerated the target in the opposite direction until it returned to the screen where it had come from (see Fig. 2A). After the target had been moved between screens for the required number of times, subjects received a food reward accompanied by a beep sound. If the target was not decelerated by touching it after it had moved from one screen to another, it eventually disappeared beyond the outer screen border, and the trial was over and no food reward was provided.

All subjects went through a staircase training procedure where the task was incrementally made more difficult by increasing the speed of the target and the number of times it needed to go from one screen to another to gain a reward (range 1–3 inter-screen crossings). At the beginning of the training each touch accelerated or decelerated the target by 5.89 cm/s, at the end of the training each touch accelerated the target by 11.8 cm/s. Subjects were encouraged by the experimenter to re-engage with the apparatus during periods of inactivity. After both subjects designated as a pair had managed to individually complete at least 75% percent of trials within a session at the last stage of training (matched in difficulty to regular Joint trials, see below), they moved to the Joint Training phase.

In the Joint Training phase, each of the two screens was installed in one of two adjacent rooms, which were connected through a sliding door. The configuration of the rooms allowed subjects to observe each other while working at their respective screens (Supplementary Video S02 and Fig. 1). A trial could be initiated from any of the two screens, and to complete it subjects had to send the target from one screen to another four times. One of two experimenters was assigned to each subject and upon success, each subject received a food reward from the respective experimenter. In some sessions the door between the two rooms was open allowing free roaming during the experiment, in other sessions it was closed (for the exact number of sessions each dyad received see Supplementary Table S2C). When the door was open, the layout of the testing room made it nearly impossible for one subject to complete a trial by moving between the two screens. To maintain subjects’ motivation we simplified the task after repeated failure: If subjects did not complete three trials in a row, the target stopped at the outer screen border in the ensuing trial. Thus, if subjects kept interacting with the target it was guaranteed to bring a reward. After Joint Training subjects moved to the testing phase. Because of sizable differences between pairs with regard to maximum performance and day-to-day variability in performance, different numbers of training sessions were administered to the three pairs in this group. For details, see supplementary materials.

#### Group B

A second group of subjects (see group composition in Supplementary Table S3A) was initially trained to do the task jointly from the very beginning. These subjects went through the same stair-case training procedure as the first group during Individual Training, except that minimum performance criteria were applied to dyadic performance in a session. All sessions were conducted with the door closed. The last stage of training was matched in difficulty to the Joint Training sessions for Group A. After a dyad successfully completed at least 75% of trials at the last stage of training, subjects moved to the Individual Training, where they performed the task alone. The set-up during these sessions was identical to that during Individual Training sessions for Group A. The difficulty of trials was matched to the last stage of Joint Training, that is, the target speed was 11.8 cm/s, and a completed trial required the target to be sent to another screen four times. After both subjects from a pair succeeded in least 75% of trials during a session, they moved to the testing phase.

#### Testing phase, both groups

In the Joint Test we included probe trials that simulated a coordination breakdown. Four probe trials were randomly dispersed among four blocks of regular trials, 20 trials in total, with the constraint that a probe trial could not occupy the first or the last position in a block of five trials. The probe trials included two types of probe events corresponding to two experimental conditions: *No Target* and *Frozen Target*, as described in the introduction. In the *No Target* condition the target did not appear on the screen it had moved towards. This manipulation was intended to create situations in which both partners stayed inactive, a situation hypothesized to make the “receiver” of the (now disappeared) ball appear *unwilling* to return it. In the *Frozen Target* condition the target stopped at the inner border of the screen it had moved towards (in the white region) after it had left the other screen and became unresponsive to touches. Subjects experiencing the Frozen Target on their screen were expected to keep touching the target, giving the impression of being willing but *unable* to return the target. Importantly, because of a restricted viewing angle through the side panels, a subject working on one screen could not see the inner part of the other screen. This ensured that the subject who had moved the target to the screen where either a *Frozen Target* or a *No Target* event occurred, could not observe the presence or absence of the target directly (see Supplementary Video S03 for an example of a Frozen Target trial), but could only infer it on the basis of their partner’s behavior (touching vs. not touching their screen). The probe events were presented with short (15 s) and long (30 s) durations. After the end of a probe event the inter-trial blank screen appeared on the display. Subjects received no food reward in these trials.

We manipulated visibility of the partner by installing opaque occluders into the side windows (see Fig. 1) in half of the test sessions, and transparent plexiglass panels in their place in the rest of the sessions. Occluders were installed at the beginning of a session in subjects’ presence and prevented direct visual access to the partner and its working area. Thus, in these sessions subjects were aware that a partner was there, but would not see them throughout the session without additional effort. The rationale for this manipulation was to investigate whether subjects would actively seek information about why coordination has broken down (e.g. by stretching and changing one’s position to get a better view at their partner). We predicted higher rates of such checking behavior in test trials with the blocked view compared to trials with unobstructed view of the partner’s workspace. Since our observations of instances of checking behavior did not reach conventional levels of acceptable interobserver agreement (see Supplementary Information), we do not report their analysis.

Within a Joint Test session each subject received one *No Target* probe trial and one *Frozen Target* probe trial. All pairs, except for the pairs Kara – Fraukje and Tai – Sandra, were tested with a closed door between the compartments. Where subjects were allowed to roam in the testing room and to swap their position between the two different screens, the experimenter tracked the subjects’ positions and made sure that both subjects received equal amount of probe events of both types. This was achieved in all sessions except for one session involving Tai - Sandra, where one of the subjects received three probe events.

In the Individual Test sessions, the setup was identical to joint testing sessions, that is, the two screens were installed in the adjacent testing rooms (as in Fig. 1), but subjects performed the task alone. Note that this is different from the individual training condition, in which the subject had both screens available to them in a single room. To complete an individual regular test trial, subjects merely needed to send the target from their directly available screen to the other. These trials served purely to motivate the subjects to interact with the task. They received the reward as soon as the target had completely left their screen. Two experimenters were present in the testing room (similar to Joint trials), each at one of the displays, ready to provide the reward. Four probe trials were randomly dispersed across four blocks of trials in a session. These probe trials included two types of probe events – *Slow Target* and *No Target*, serving as control conditions for the Joint Test. In the *Slow Target* probe event the target appeared on another screen, and slowly moved in the direction of the subject’s screen. The speed of the Slow Target corresponded approximately to the average time a target was present on one screen before switching to another screen for that dyad in regular Joint Practice trials. As soon as the target fully entered their screen, subjects received the reward. These trials thus simulated a situation where subjects had to wait for the target to return during regular Joint trials, but without the other subject in fact being present.

In the *No Target* trials the target did not appear on the other screen after it had left the subject’s screen. The No Target trials lasted 15 s or 30 s to match the Joint Test probe trials. The No Target trials were designed to be compared with the Joint No Target probe trials but without another subject being present.

Subjects received two probe trials of each type (No Target and Slow Target) during a session and received no food reward after their completion. The position of the probe trials within a session was subject to the same constraints as in the Joint Test. Half of the Individual Test sessions were run with transparent side panels where subjects, and the other half was run with opaque side panels (see Fig. 1). In both conditions subjects were aware that there was no partner at the second screen because opaque panels were inserted after the experiment started.

### Design

**Figure 5:**
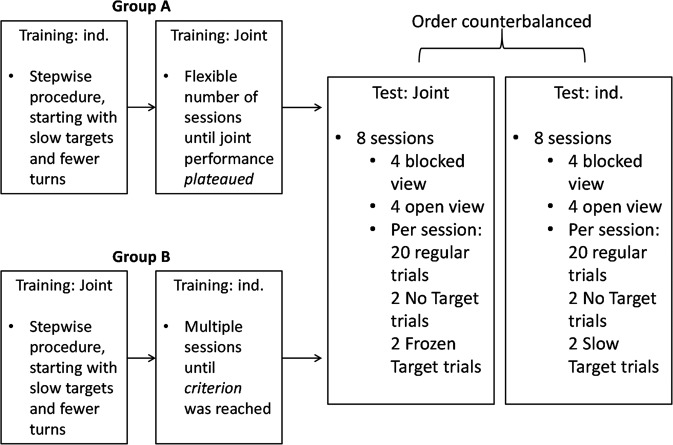
A flow-chart with the consecutive sequence of experimental phases.

Individual/Joint Training, Individual/Joint Practice, and Individual/Joint Test phases were run in a blocked design with trials grouped into sessions (see also Fig. 5 with a flow-chart illustration of the design). Each session included a total of 20 trials. At the beginning of each session, subjects received four additional warm-up trials which they could not fail because the target did not leave the screen even if it was not touched.

Each pair and each subject (except for Kara, who did not complete the Individual Test, see Supplementary Information) completed eight Joint Test sessions and eight Individual Test sessions. Fraukje completed a second set of eight Individual Test sessions because she was paired with two different partners (Kara and Robert). Within each pair, one subject, chosen randomly, completed the Individual Test block before the Joint Test block; for the other subject the order was reversed. Visibility of the partner and duration of probe events in Joint Test and Individual Test were varied between sessions: Two sessions were conducted for each combination of the two factors. Tai and Sandra first completed two sessions with blocked view in both Social and Individual Tests. All other subjects completed sessions with open view prior to exposure to blocked view in both tests.

### Behavior coding

Subjects’ behavior during task performance was coded off-line for critical gestures using a pre-defined list that included e.g. clapping, banging on the side panels, fingers in the panel hole and mouth gestures (see Supplementary Video S04 for examples and Table S1 for a full list). Any *visual* gesture (e.g. pointing) was only coded as such if it was directed towards the partner (in the Joint Test and training conditions) or if it was directed towards the location in which the partner would normally be (in the Individual Control condition). Conversely, auditory gestures (e.g. clapping) were coded regardless of whether they were directed to the (potential) partner’s location, because an auditory signal does not need to be seen by its addressee in order to be received. The categories in the list and their operational definitions were chosen based on previous studies that investigated communicative behavior in chimpanzees and other non-human great apes^34,60–62^. Warm-up trials and motivation trials were not included in the analysis of behaviors.

A second coder coded a randomly chosen set of 32 sessions from the pool of all available joint training sessions with regard to whether a gesture was observed within a given turn or not, individual test and joint test sessions. Interrater reliability was satisfactory with kappa = 0.771, a number not atypical for studies of primate gestures^27,29,72^. For more details, see Supplementaries.

### Statistical analyses

To address our first main question – whether chimpanzees would use gestures to maintain coordination in the face of a breakdown – we compared the frequency of such behaviors in regular vs. probe turns of the Joint Test trials. This analysis included data from the Joint Test sessions only. For statistical inference on the individual level, we fitted generalized linear models with binomial error structure and logit link function (R package lme4, function glm) that predicted occurrence of at least one gesture in a given turn as a function of turn type (regular vs. probe), trial (within session), session (within view condition), view condition (blocked vs. open). Statistical inference was conducted using likelihood ratio tests, comparing the full model with a reduced model that did not include turn type as a factor (R package lme4, function drop1). Because of instability of models for those two subjects who never displayed gestures in regular turns (which resulted in convergence issues and/or inflated parameter and standard error estimates), the sensitivity of results was assessed by also analyzing the datasets using Firth logistic regression (R package logistf, function logistf), a method that uses penalized likelihood estimation and which is less vulnerable to complete separation issues than standard GLMs^73^ (see also Supplementary Information) as well as traditional Χ^2^-tests of independence (disregarding control variables). Results of these different analyses yielded the same conclusion in almost all cases. Table 2 shows the results of Χ^2^-tests of independence and Firth logistic regression; detailed results of GLMs can be found in the Supplementary Information. To assess whether the chimpanzees gestured more frequently in the probe trials at the group level, two generalized linear mixed models (GLMMs) were fitted (R package lme4, function glmer), each of which predicted occurrence of at least one gesture in a given turn as a function of turn type (regular vs. probe), trial (within session), session (within view condition), view condition (blocked vs. open), and turn duration. Each of the two models excluded one of the pairs that shared a subject. The parameter estimates for the full models can be found in Supplementary Table S5A. Both models were stable, that is, the exclusion of any specific subject did not change the sign of the effect of turn type.

To address our second main question – whether chimpanzees would use gestures more frequently when playing with a partner than when playing alone – we completed two main analyses. In a first step, we compared the frequency of turns with gestures between the Joint No Target probe trials and the Individual No Target probe trials. For each subject, we fitted one or more generalized linear models to predict gestures in a given turn as a function of condition (individual vs. joint test), trial (ranging between 1 and 24), session within view block (1–4), and view (blocked vs. open). Statistical significance of the effect of condition was assessed using likelihood ratio tests. In addition, Χ^2^-test were computed for the simple cross-tabulation of condition and gestures on the individual level, disregarding potential effects of session, trial and view condition. We again fitted for each subject Firth logistic regression models to assess the sensitivity of results. For details of these analyses, see Table 3 and Supplementary Table S6. Analogously to the first set of analyses, we fitted two generalized linear mixed models to assess the effect of joint vs. individual testing on gesturing on the group level. Model details can be found in Table S5B. As a second comparison to test the hypothesis that the selected communicative behaviors are more frequent when a partner is present, we compared the frequency of turns that included at least one gesture between turns in Joint regular trials and in Individual Slow Target trials.

## Supplementary information

Supplementary Video S01.

Supplementary Video S02.

Supplementary Video S03.

Supplementary Video S04.

Supplementary Information.

## Data Availability

The data that support the findings of this study are available from both corresponding authors upon request.
