# Supplementary material for: Chimpanzee Coordination and Potential Communication in a Two-touchscreen Turn-taking Game
Source: Sci Rep. 2020 Feb 25;10:3400. doi: 10.1038/s41598-020-60307-9 (PMC7042301; doi:10.1038/s41598-020-60307-9)
Supplement: Supplementary file 5 — Supplementary Information. [file 41598_2020_60307_MOESM5_ESM.pdf]

Chimpanzee Coordination and Potential Communication in a Two-touchscreen Turn-taking game

Pavel V. Voinov[1], Josep Call[2,3], Günther Knoblich[1], Marina Oshkina[1], and Matthias Allritz[2,3]

[1] Department of Cognitive Science, Central European University, Oktober 6 u. 7, H-1051 Budapest, Hungary

[2] Department of Developmental and Comparative Psychology, Max Planck Institute for Evolutionary Anthropology, Deutscher Platz 6, Leipzig D-04103, Germany.

[3] School of Psychology & Neuroscience, University of St Andrews, St. Andrews, Fife KY16 9JU, UK.

Supplementary Information

Gesture coding

Table S1. Gestures coded in Joint Training, Joint Test and Individual Test conditions.

Gesture	Definition
Visual Gestures	
Arm fling	Rapid movement of the hand or arm in the direction of the partner
Sweeping gesture	Subject waves with its arm/hand in a sliding motion
Hand beg	Subject puts finger(s) or hand through the mesh grid with the palm upwards
Pointing	Subject pokes (or tries to) and retrieves his finger(s) or hand through the mesh grid or the test panel with the palm downwards
Mouth gestures	Mouth on hole / tongue in hole<br>Subject puts open mouth or tongue into the hole of the test panel<br>Mouth on panel<br>Subject puts open mouth on the test panel (not in the hole)
Auditory gestures	
Hand Clap	Strike an open hand or other part of body with hand and thereby producing an audible noise. Continuous clapping is considered as one instance unless there is at least a 2 s. interval between two claps
Banging	Banging: Subject makes noise by striking the floor or glass walls with hand, palm or foot
Knocking	Knocking Subject knocks at the panel or mesh with its knuckles, back of the hand or wrist
Flip panel	Subject puts its finger in the hole of the test panel and rocks it

Visual gestures were coded only if the gesture was made (= arm, hand or mouth was pointing) in the direction of the partner, or, in case of the individual test condition, in the direction of the area in front of the of the second touch screen. Auditory gestures were coded regardless of directedness.

Interrater reliability was established, for all gestures combined, in the following manner. A second coder coded a randomly chosen set of 32 sessions from the pool of all available joint training, individual test and joint test sessions. For each of these sessions, it was determined for each of the two coders whether they saw an audible or visible gesture for a given subject in a given turn, yielding a total of 4202 events for which each coder provided an observation. Based on these 4202

events, interrater reliability was determined to be satisfactory (κ = .771). The reliability sample was representative of the dataset as a whole in that the frequency of events coded by coder 1 as "turn included gesture" (198 of 4202, or 4.71%) mirrored the low average frequency of communication in the dataset as a whole.

Note that for all statistical analyses of gesturing during joint training (see Table 1 in main text), only those training stages were considered in which subjects played across two rooms (see Tables S2C and S3B below). This was done to allow for comparability between training groups (Individual Training First and Joint Training First) and between training and test conditions (the latter were only run with the two-room setup).

Training Procedure

Table S2A. Subjects of group "Individual Training First"

Individual trained	Sex	Age
Fraukje	F	39
Kara	F	10
Tai	F	13
Sandra	F	22
Robert	M	39
Riet	F	37
Dorien*	F	34

* Training was not completed because subject lost interest in the task and failed to complete a full session over the course of multiple attempts

Table S2B. Pair composition of group "Individual Training First"

Pair trained	
Fraukje	Kara*
Fraukje	Robert
Tai	Sandra
Riet**	Sandra**
Riet**	Tai**

*Kara did not complete the Individual Test condition because the subject had to be transferred to another facility before the condition was administered.

** Training was not completed because one subject lost interest in the task and failed to complete a full session over the course of multiple attempts.

Table S2C. Staircase training procedure for group “Individual Training First”

**Training Stage**	**Step**	**Training situation**	**Target speed**	**Required screen crossings**	**Failure possible**	**Number of sessions**
Individual	1	One room, individual	1/2x	2	No	1-4
Individual	2	One room, individual	1/2x	2	Yes	1-23
Individual	3	One room, individual	3/4x	2	Yes	1-13
Individual	4	One room, individual	1x	2	Yes	1-3
Individual	5	One room, individual	1x	3	Yes	1-22
Individual	6	One room, individual	1x	4	Yes	1-13
Joint	1	One room, social screens adjacent	1x	4	Yes	2
Joint	2	One room, social screens at 90° angle[1]	1x	4	Yes	2-7
Joint	3	Two rooms, social door between rooms open	1x	4	Yes	7-16
Joint	4	Two rooms, social door between rooms closed	1x	4	Yes	2-29

[1] One pair (Fraukje-Kara) received an additional seven sessions with the 90° angle setup (both screens installed in the same room, with one screen installed in place of a side panel, see Figure 1) as part of a piloting procedure (see below). These sessions incrementally increased in difficulty (starting with slower target and fewer screen crossings required) similar to this pair’s Individual Training procedure. In total, this pair completed 14 sessions in the 90° angle training condition.

It should be noted that for the group who received joint training after the individual training phase, two additional joint training setups were attempted (Steps 1 and 2 in the Joint Training stage in Table S2C). Because these three pairs were considered highly socially tolerant of each other, they were first tested in two setups where both touch screens were installed in the same testing room. As these setups proved inappropriate for the purpose of joint performance assessment (often both screens would be monopolized by the more dominant subject), this was later abandoned in favour of testing subjects jointly *across two rooms* only, and only data from those sessions are included in all analyses of Joint Training.

Table S3A. Pairs composition of group "Joint Training First"

	Individual trained	Sex	Age
Pair 1	Lobo	M	12
	Kofi	M	11
Pair 2	Lome	M	15
	Bangolo	M	7

Table S3B. Staircase training procedure for group "Joint Training First"

Training Stage	Step	Training situation	Target speed	Required screen crossings	Failure possible	Number of sessions
Joint	0	Two rooms, social door between rooms closed	1/2x	2	No	1
Joint	1	Two rooms, social door between rooms closed	1/2x	2	Yes	1-3
Joint	2	Two rooms, social door between rooms closed	3/4x	2	Yes	1
Joint	3	Two rooms, social door between rooms closed	1x	2	Yes	2-7
Joint	4	Two rooms, social door between rooms closed	1x	3	Yes	1-9
Joint	5	Two rooms, social door between rooms closed	1x	4	Yes	1-11
Individual	1	One room, adjacent screens	1x	4	Yes	3-22

Note. 1x target speed was equal to 11.8 cm/s.

Training Performance

Table S4A presents the mean individual success rates for all subjects across the last five sessions of individual training, unless subjects required fewer sessions to pass. Individual performance on warmup trials was not considered. Interspersed trials in which the computer automatically stopped the target at an outer screen edge to give subjects more time to respond (see Methods) were counted as unsuccessful. The criterion for ending individual training was the same for all individuals (first session that required four turns per trial in which the subject completed 15 or more trials successfully). The mean success rate for individual subjects ranged from 56% to 85%, with the exception of two subjects (males Bangolo and Kofi) who reached criterion very abruptly after two to four sessions with success rates of 20% or less.

Table S4A: Mean success rate and range across last five sessions of individual training

Individual	Sex	Age	Group	Total number of training sessions	Mean success rate and range across last five sessions (in %)	
Fraukje	F	39	Individual Training First	12	73	45 – 95
Kara	F	10	Individual Training First	9	85	65 – 95
Tai	F	13	Individual Training First	7	78	65 – 90
Sandra	F	22	Individual Training First	10	84	75 – 95
Robert	M	39	Individual Training First	51	56	30 – 85
Lome	M	15	Joint Training First	3	58.33*	35 – 85*
Bangolo	M	6	Joint Training First	5	21	0 – 85
Lobo	M	11	Joint Training First	22	64	55 – 80
Kofi	M	10	Joint Training First	3	35*	0 – 85*

Note. Number of required turns was not equal for all of the last five sessions for all individuals

* values for three sessions only

Two sessions were repeated because a subject initiated only one trial and was reluctant to proceed. These aborted sessions were not included in statistical analysis.

Table S4B presents the mean joint success rate (same trial inclusion criteria as for individual success rate applied) for all pairs across the last five sessions of joint training. For those three pairs who completed the joint training condition after having already completed the individual practice condition, criteria for ending practice were determined ad hoc, rather than being identical for all subjects. This was done because some subject pairs showed very different degrees of day-to-day variability in their joint success rate (see Fig S1). For the other two pairs (those who started with the joint practice condition), the same end-of-training criterion was applied that had been used in the case of individual training (training ended after the first session that required four turns per trial in which the subjects jointly completed 15 or more trials successfully). By the end of joint training, the mean joint success rate ranged between 44 and 92 percent across the five pairs.

Table S4B. Mean success rate and range across last five sessions of joint training

Pair	Order of training	Total number of training sessions	Mean success rate and range across last five sessions (in %)	
Fraukje - Kara	joint second (4 turns)	10 (26)	92	80 – 100*
Fraukje - Robert	joint second (4 turns)	36 (40)	53	30 – 80
Sandra – Tai	joint second (4 turns)	18 (22)	54	15 – 75*
Lome – Bangolo	joint first (staircase)	24	44	10 – 80
Lobo - Kofi	joint first (staircase)	15	64	40 – 95**

Note: for the numbers of training sessions, values in parentheses indicate number of training sessions including those conditions that were not completed by all subjects (see below).

* the two last sessions were completed with door closed between cages

** Number of required turns was not equal for all of the last five sessions for this pair

Note also that the first three pairs in Table S4B (the group that completed Individual Training first) received additional training conditions as outlined in Table S2C. Session totals in parentheses include these conditions, numbers outside parentheses include only those conditions in which the game was played across two rooms and which were included in the statistical analyses of Joint Training.

During five of the joint training sessions played across two rooms, either an error occurred (e.g. screen failure), or the subjects did not complete the session. Such sessions were repeated and only the repeated session was included in the analyzed data.

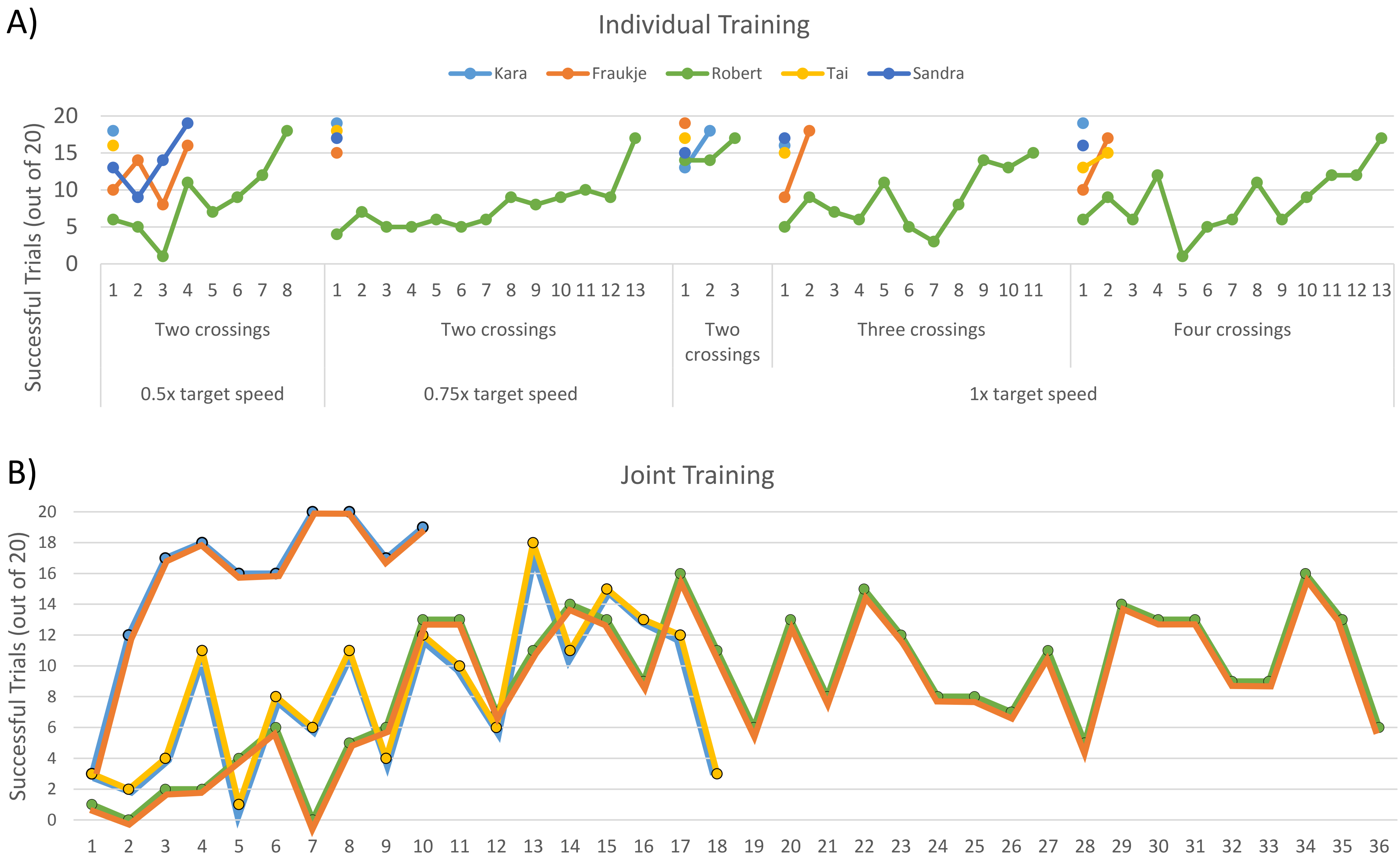

Figure S1. Performance of pairs that were trained individually first plotted against sessions. A) Individual Training phase. B) Joint Practice phase. Target speed refers to the speed a stationary target would gain upon a single touch. 1x target speed was equal to 11.8 cm/s.

A)

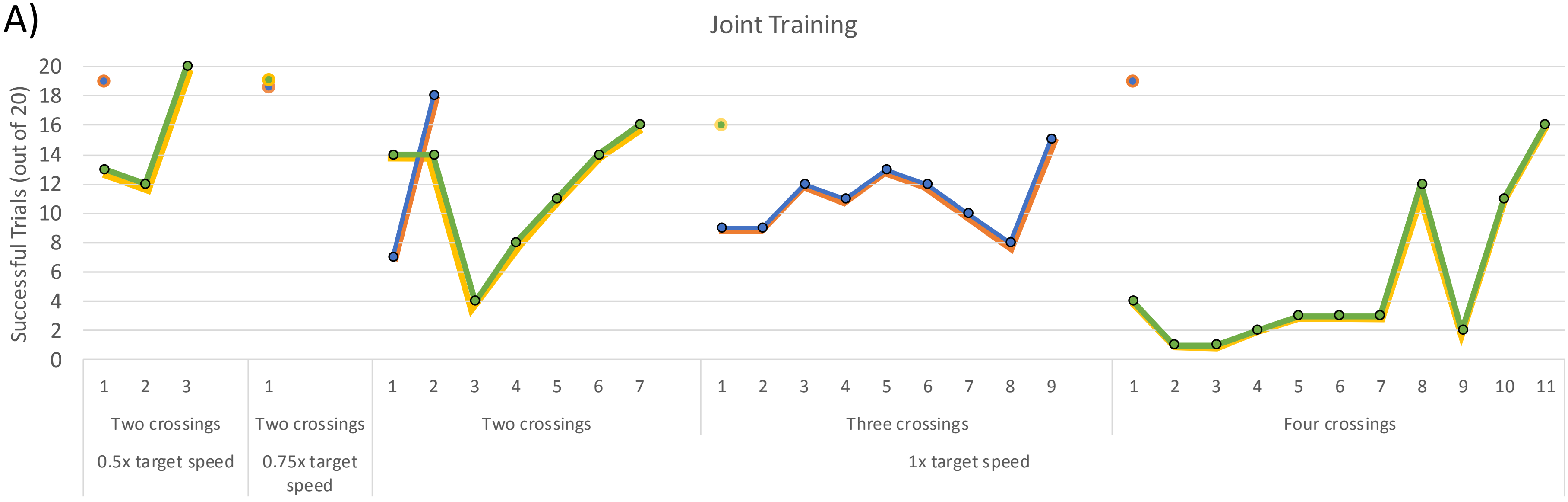

B)

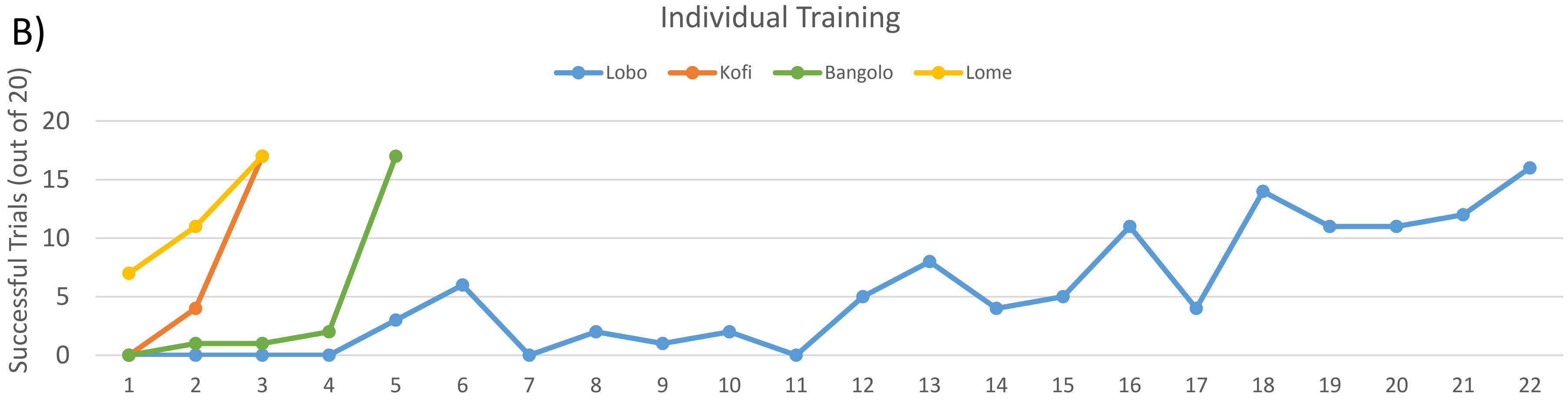

Figure S2 Performance of pairs that were jointly trained first plotted against sessions. A) Joint Training phase. B) Individual Practice phase. At the second stage of Joint Training both pairs successfully completed 19 trials (individual markers are displaced for visualization purpose).

Full model outputs for group level models

Gesturing in regular vs. probe turns of the Joint Test trials

Table S5A. GLMM results for the group level comparison of gesturing between regular and probe turns in the Joint Test condition

Predictor	β	*SE*	X^2	*p*
Model excluding pair "Fraukje - Kara"				
turn type: probe	2.05	0.50	6.34	.012
trial (within session)	0.17	0.20	0.72	.398
session (within view condition)	-0.14	0.22	0.50	.481
duration	0.63	0.24	5.09	.024
view: open	-0.33	0.77	0.16	.691
Model excluding pair "Fraukje - Robert"				
turn type: probe	1.92	0.47	6.50	.011
trial (within session)	0.06	0.16	0.12	.728
session (within view condition)	-0.09	0.18	0.23	.634
duration	0.61	0.20	6.58	.010
view: open	-0.10	0.59	0.03	.868

Note: continuous predictors (trial, session, duration) were standardized.
Reference levels: turn type – regular, view – blocked.

Gesturing in No Target probe turns of the Joint vs. Individual Test trials

Table S5B. GLMM results for the group level comparison of gesturing in Joint Test vs. Individual Test No-Target probe turns

Predictor	β	*SE*	X^2	*p*
Model excluding pair "Fraukje - Kara"				
condition: Joint Test	1.12	0.82	1.60	.205
trial (within session)	-0.01	0.27	0.00	.980
session (within view condition)	0.55	0.20	6.41	.011
view: open	0.11	0.47	0.05	.831
Model excluding pair "Fraukje - Robert"				
condition: Joint Test	1.35	0.79	2.35	.125
trial (within session)	-0.20	0.34	0.32	.569
session (within view condition)	0.43	0.20	4.43	.035
view: open	0.53	0.39	1.77	.183

Note: continuous predictors (trial, session) were standardized.
Reference levels: condition – Individual Test, view – blocked.

Sensitivity analyses: GLM fitted with maximum likelihood estimation

Gesturing in regular vs. probe turns of the Joint Test trials

Table S6 is identical to Table 2 from the main text, with the addition of results from an analysis with traditional maximum likelihood parameter estimation using the R package lme4. For each individual, we fitted a generalized linear model with binomial error structure and logit link function (R package `lme4`, function `glm`) to predict gesturing in a given turn as a function of condition (individual vs. joint), view (blocked vs. open), session within view block (1-4), and trial (ranging between 5 and 24 because warmup trials were excluded). All continuous variables (in this case session and trial) were standardized before model fitting. Statistical significance was assessed using likelihood ratio tests (LRT), comparing the full model with a reduced model that did not include condition as a factor, using the function `drop1` from the `lme4` package.

Table S6. Differences in gesturing frequency between Joint Test regular turns and Joint Test probe turns

	Sign of	Firth logistic regression				Maximum likelihood estimation				X^2-test	
	difference	β	*SE*	X^2	*p*	β	*SE*	X^2	*p*	X^2	*p*
Kara	+	4.28	0.74	31.68	< .001	4.55	0.83	31.12	< .001	76.37	< .001
Fraukje*	+	3.33	0.55	28.44	< .001	3.42	0.58	27.34	< .001	65.95	< .001
Fraukje**	+	4.26	1.23	11	0.001	†	†	†	†	19.04	< .001
Robert	+	3.86	1.27	6.26	0.012	†	†	†	†	3.9	0.048
Sandra	+	3.03	0.49	36.24	< .001	3.12	0.5	36.03	< .001	56.26	< .001
Tai	+	1.6	0.53	7.88	0.005	1.59	0.54	7.25	0.007	5.35	0.021
Bangolo	+	4.69	0.77	43.02	< .001	5.09	0.9	43.37	< .001	103.21	< .001
Lome	+	3.44	0.52	39.09	< .001	3.55	0.54	38.53	< .001	82.14	< .001
Lobo	+	4.42	0.78	38.52	< .001	4.84	0.92	39.29	< .001	88.43	< .001
Kofi	+	2.15	0.97	3.15	0.076	2.12	1.28	2.06	0.151	0.64	0.423

* as paired with Kara, ** as paired with Robert

† ML estimates excluded because of model instability

Gestures in Joint vs. Individual Test conditions

Table S7 is identical to Table 3 from the main text, with the addition of results from an analysis with maximum likelihood parameter estimation using the R package lme4. The complete lack of gesturing events in the individual condition for four subjects (male Lobo and females Sandra, Tai and Fraukje, as paired with Robert), accompanied by model instability and / or extreme values for beta and standard error estimates pointed to a complete separation problem (Heinze & Schemper, 2002). In addition to Firth logistic regression and traditional (non-parametric) X^2-association tests, as presented in the main text, the second set of columns in Table S7 presents the results of a replacement procedure that has been used occasionally to treat problems of complete separation (e.g. Wilson et al., 2014). For each of the four subjects for whom models did not converge using the `glm` function in `lme4`, 16 additional models were fitted with the same model formula for an identical dataset in which each one of the 16 zeros from Individual Test trials was replaced with 1, one at a time. Models based on these dummy datasets may be considered more conservative than the original model, as each of them contains one extra data point not in favor of the main hypothesis. Ranges for parameter and standard error estimates for these more conservative models are presented along with minimum X^2 and maximum *p*-values for those subjects whose data was affected by the complete separation problem.

Table S7. Differences in gesturing frequency between Joint and Individual Test condition in No Target probe turns

	Sign of	Firth logistic regression				ML / Replacement Procedure†				X^2-test	
	difference	β	*SE*	X^2	*p*	β (range)	*SE* (range)	X^2 (min)	*p* (max)	X^2	*p*
Fraukje*	+	1.03	0.97	1.3	.255	1.35	1.09	1.69	.194	0.21	.651
Fraukje**	+	1.63	1.4	1.41	.236	0.60 – 1.72	1.3 – 1.69	0.19	.660	0.53	.465
Robert	-	-1.54	1.14	2.15	.143	-2.21	1.45	2.96	.085	0.95	.330
Sandra	+	3.46	1.52	9.43	.002	2.68 – 3.36	1.21 – 1.47	6.46	.011	8.17	.004
Tai	+	2.73	1.75	3.46	.063	††	††	††	††	1.47	.225
Bangolo	-	-2.06	1.02	5.51	.019	-2.68	1.22	6.98	.008	2.07	.150
Lome	+	0.78	0.8	1.1	.295	0.94	0.84	1.3	.253	0.62	.432
Lobo	+	4.02	1.86	8.59	.003	2.37 – 5.35	1.22 – 2.55	5.13	.023	5.13	.024

Table S6. Differences in gesturing frequency between Joint and Individual Test condition in No Target probe turns

* as paired with Kara, ** as paired with Robert

† replacement procedure applied and ranges reported only if full or null model based on original data did not converge with maximum likelihood estimation

†† results excluded because some of the replacement models remained unstable

Comparison of probe trial types in Joint Test condition

We further investigated whether the rate of gesturing differed between the Frozen Target and No Target conditions for the senders of the probe event and the receiver. These differences are illustrated in Figure S3.

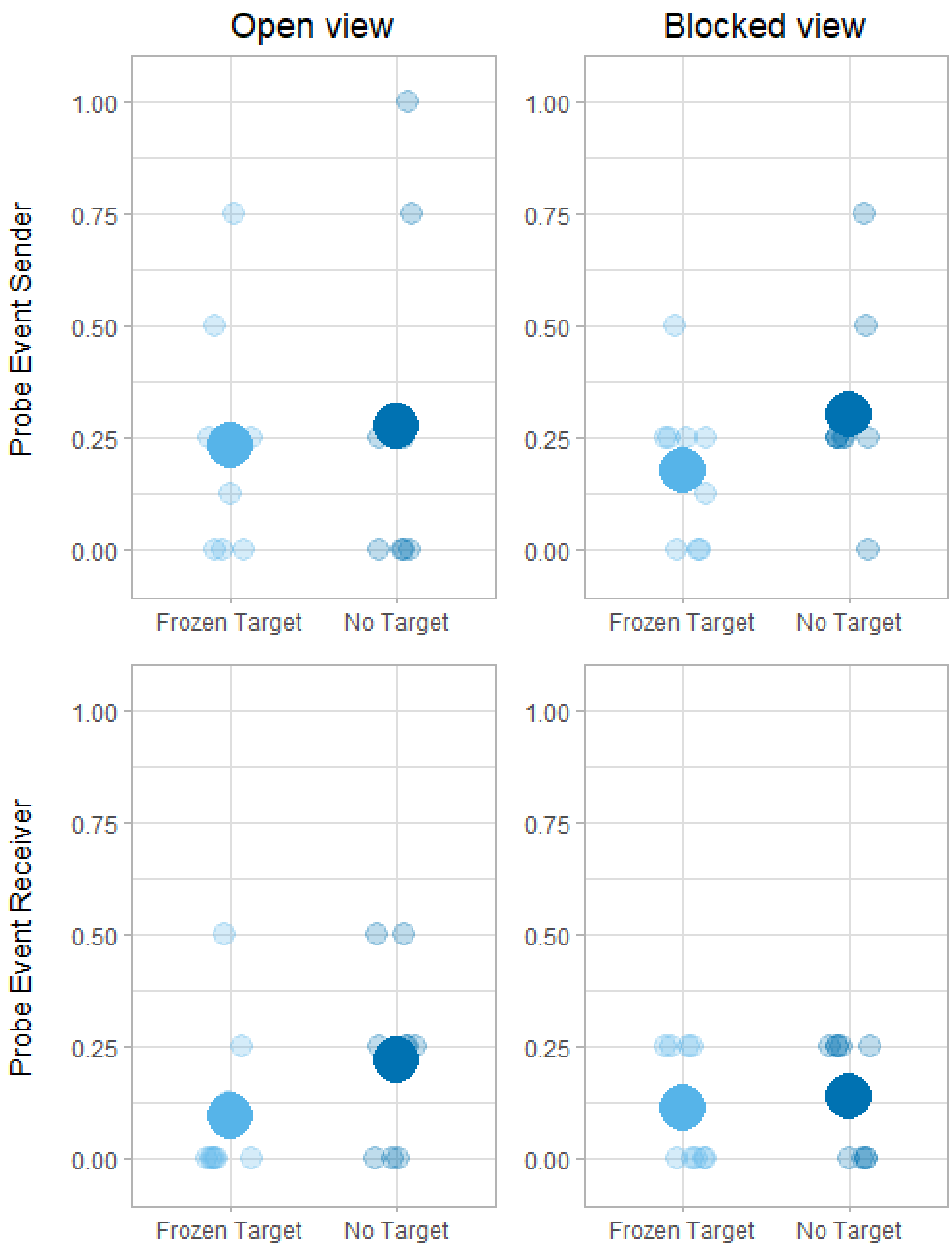

Fig S3. Proportion of turns with gesturing for different probe types in the Joint Test condition. Small dots depict individual data points, large dots depict group means.

We conducted separate analyses based on whether the subject was the "sender" or the "receiver" of a probe event. We hypothesized that if a subject *sent* the target to the other side and it ended up

frozen on the partner's screen, this would in turn prompt the partner to touch that screen (more specifically, the target) more frequently (appearing willing but "unable" to the subject, see Methods) than if the trial was a No Target probe trial ("unwilling" condition). A manipulation check showed that for all receivers this was indeed the case: receivers touched the screen more often in Frozen Target trials (mean rate of touching the screen 1.31 touch/s, *SD* = 0.805) than in No Target (*M* = 0.78 touch/s, *SD* = 0.698). We fit a Mixed Linear Model (SPSS MIXED procedure) with "Probe Trial type" (Frozen Target vs. No Target) as a fixed factor and "Subject" as a random factor with random intercepts and random slopes. The effect of Trial Type was found to be significant, $F(1, 155.054) = 35.138$, $p < .001$. We further hypothesized that a sender of the probe event, if they were sensitive to the difference between an unwilling and an unable partner, would communicate more frequently in cases in which their partner did not touch the screen (that is, in turns with No Target) than if their partner did. Finally, we expected that this difference should be amplified in, or even be exclusive to, cases in which the sender could actually see their partner's efforts (or lack thereof), that is in the open view condition. When modelling sender behavior we thus expected a two-way interaction between probe trial type and view condition. Because of the low number of trials per combination of conditions, for this analysis and the analysis of receiver behavior, no inferential statistics were computed on the level of individual subjects. For the group, multiple GLMMs were fitted to assess whether the predicted interaction existed. In a first step, a full model was fitted which included as fixed effects "probe type", "view", their interaction, as well as "session" and "trial", and the random effect "subject" with an intercept term and all possible random slopes associated with it. This model was compared via likelihood ratio test (R package `lme4`, function `anova`) to a reduced model that included the same terms except the fixed effect terms "probe type", "view" and their interaction ("full-null-model comparison"). As in previous analyses, two separate groups of models were fitted, each one excluding in the underlying dataset one of the two pairs that shared a subject.

As can be seen in the top row of Figure S3, for the subject that last sent the target towards their partner before a probe event occurred, the prediction of an interaction that implies an amplified effect of probe type in the open view condition was not confirmed by the data: while a small difference between probe types appeared to be present in both view conditions, if anything that effect was stronger in the blocked condition (proportion of turns with gesturing in turns with No Target: *M* = 0.31, *SD* = 0.21; Frozen Target: *M* = 0.18, *SD* = 0.17), rather than the open view condition (No Target: *M* = 0.28, *SD* = 0.36; Frozen Target: *M* = 0.24, *SD* = 0.25). The full-null model comparison revealed a non-significant difference between models (Model 1, excluding pair "Fraukje & Kara": $X^2(3) = 3.81$, $p = .282$; Model 2, excluding pair "Fraukje & Robert": $X^2(3) = 0.79$, $p = .851$). Thus for chimpanzees who were probe senders, the predictors "view", "probe type" and their

interaction, considered in conjunction, did not significantly improve the prediction of gesturing behavior. Models fitted on the basis of dataset 1 were stable in the sense that the exclusion of a specific subject did not change the (negative) sign of the interaction between probe type and view condition, whereas for models fitted with dataset 2 (excluding pair “Fraukje-Robert”) the interaction estimate even changed sign when male subject Bangolo was excluded from model fitting.

With regard to the *receiver* of probe events, the predictions were more straightforward: irrespective of whether partners can see each other, the receiver of a No Target event, who is effectively staring at an empty screen for up to 30 seconds, should be more inclined to communicate with their partner than if they receive a Frozen Target (which instead turns the responsibility on them to continue the game). Two GLMMs were fitted to assess the effect of probe type among probe target receivers. These models included as fixed effects “probe type” (as the only test predictor), “session”, “trial” and “view” as control predictors and “subject” as random effect. All of these models were stable; the exclusion of a specific subject did not change the sign of the estimated effect of probe type. As can be seen in the bottom row of Figure S3, there was indeed a tendency for the receivers of a probe turn to communicate more when they received no target (overall proportion of turns with gesturing: $M = 0.18$, SD = 0.13) than when they received a Frozen Target (overall proportion of turns with gesturing: $M = 0.10$, $SD = 0.10$). However, on the group level this effect was not significant (Model 1, excluding pair “Fraukje & Kara”: $N = 8$, $\beta = 0.65$, $X^2(1) = 1.24$, $p = .266$; Model 2, excluding pair “Fraukje & Robert”: $N = 8$, $\beta = 0.64$, $X^2(1) = 1.51$, $p = .220$). Wilcoxon signed-rank tests that compared the proportion of turns with gesturing between the two probe types suggested the same conclusion (Dataset 1: $T = 2.5$, $N = 8$, $p = .313$, Dataset 2: $T = 2$, $N = 8$, $p = .250$).

Checking behaviours

Table S8 lists the three behaviours we included in our analysis of whether chimpanzees checked their partner's side in specific conditions. For room setup, see Figure 1 of the main text.

Table S8. Gestures coded in Joint Test and Individual Test conditions.

Checking behavior	Definition
Stretch and look	Subject stretches above the side panel and looks at partner's working area
Looking from behind the door	Subject goes behind the corner (up to and including the doorway leading to partner's room) and looks at partner's working area
*Going to partner's side**	subject enters partner's room and approaches or even touches partner's screen (only possible for pairs tested with open door)

* Only those individuals who were tested with an open door between both testing rooms (two of five pairs) were able to go to partner's side.

Checking behaviors during test were very rare for most subjects. Checking rate in the Individual Condition (only for subjects who could move between two screens) ranged between 0.0026 and 0.0677, and between 0.0013 and 0.0343 in the Joint Test condition. Interrater reliability was established for checking behaviors in the same way it was done for gestures. The frequency of turns with checking behaviors was very low within the reliability sample (1.2% for Coder 1 and 1.1% for Coder 2) and interrater reliability was less satisfactory than it was for communication behaviors (κ = .635). For this reason, we did not analyze checking behaviours.
